# Impact of Whole-Body Vibrations on Electrovibration Perception Varies with Target Stimulus Duration

**DOI:** 10.1177/00187208251326662

**Published:** 2025-04-17

**Authors:** Jan D. A. Vuik, Daan M. Pool, Celal Umut Kenanoglu, Yasemin Vardar

**Affiliations:** 1 2860 Delft University of Technology, The Netherlands

**Keywords:** tactile displays, touchscreens, haptics, psychophysics, biodynamics

## Abstract

**Objective:**

This study investigates the impact of whole-body vibrations caused by external vehicle perturbations, such as aircraft turbulence, on the perception of electrovibration displayed on touchscreens.

**Background:**

Electrovibration is a promising technology for providing tactile feedback on future touchscreens, potentially addressing usability challenges in vehicle cockpits. However, its performance under dynamic conditions, such as whole-body vibrations caused by turbulence, remains largely unexplored.

**Method:**

We measured the absolute detection thresholds of 24 human participants for short (0.2 s) and long (0.5 s) duration electrovibration stimuli displayed on a touchscreen. These measurements were taken in the absence and presence of two types of turbulence motion (Gaussian and Multisine) generated by a motion simulator. Concurrently, we recorded participants’ applied contact force and finger displacements.

**Results:**

Electrovibration stimuli displayed on vehicle cockpit touchscreens were more reliably perceived with a 0.5-s duration than a 0.2-s duration, both in the presence and absence of turbulence. Both turbulence types led to increased vibration-induced finger displacements and scan speeds in the direction of turbulence, as well as higher applied forces and force fluctuation rates. Gaussian turbulence significantly elevated perception thresholds, but only for short-duration electrovibration stimuli.

**Conclusion:**

The findings indicate that whole-body vibrations impair the perception of short-duration electrovibration stimuli, primarily due to unintentional finger movements and increased fluctuations in applied normal force.

**Application:**

Our findings offer valuable insights for the future design of touchscreens with tactile feedback in vehicle cockpits.

## Introduction

In today’s digital era, touchscreens have become indispensable and are integrated into various electronic devices like smartphones, tablets, laptops, kiosks, and digital information panels. Their presence has also surged in vehicle cockpits across automobiles, aircraft, and vessels as they offer a multitude of advantages over traditional buttons and knobs (Ahmad et al., 2018; van Zon et al., 2020). Touchscreens can effortlessly incorporate vast amounts of information, updated through reconfigurable graphical user interfaces (GUIs), without needing to rewire mechanical controls. Additionally, they foster intuitive interactions through pointing gestures, elucidating their prevalence in contemporary vehicle cockpits.

However, challenges arise with the widespread use of touchscreens in these environments. One notable issue is the absence of tactile or aural feedback. Unlike traditional knobs and buttons that offer feedback through force or sound (Wang et al., 2018), touchscreens require users to confirm actions through alternative means, such as visually checking if a button has been pressed. This lack of tangible feedback can pose safety risks, particularly when users divert their attention to the touchscreen instead of the road, potentially leading to hazardous situations in traffic.

Moreover, using touchscreens during external perturbations, such as turbulence in aircraft, bumpy roads in cars, or waves in ships, presents difficulties. These perturbations and vibrations propagate through the human body and arm, inducing unintended relative motions between the user’s finger and the screen, also called *biodynamic feedthrough* (Venrooij et al., 2013). Various studies indicate that these propagated vibrations negatively impact user performance across different input devices (Hill & Tauson, 2005; McLeod et al., 1980; Narayanamoorthy & Huzur Saran, 2011). This effect extends to touchscreens in vehicle cockpits and hinders their use (Cockburn et al., 2017; Coutts et al., 2019; Dodd et al., 2014; Khoshnewiszadeh & Pool, 2021; Liu et al., 2022). Various methods have been explored to address these challenges, including larger buttons, increased spacing, visual and auditory feedback, and additional physical features on the screen. However, none has provided a complete solution to this problem (Coutts et al., 2019; Dodd et al., 2014; Lancaster et al., 2011; Liu et al., 2022; Wan et al., 2017).

Surface haptics, specifically electrovibration, emerges as a potential solution to mitigate the decrease in task performance during external perturbations. Electrovibration modulates perceived friction through induced electrostatic forces between a finger and a high-voltage supplied capacitive touchscreen (Bau et al., 2010); see Supplemental Materials for technical details. This technology has shown positive effects on touchscreen user performance, enhancing accuracy, efficiency, and task completion times during pan gestures and dragging tasks (Liu et al., 2018; Zhang & Harrison, 2015). Exploring its application in button or ridge rendering could improve task performance, as demonstrated in earlier work conducted with other types of surface haptic displays (Bernard et al., 2022).

Despite its potential advantages for improving touchscreen interactions in vehicle cockpits, little is known about how electrovibration technology can be utilized during external vehicle perturbations (e.g., sudden impacts, bumpy roads, waves, and turbulence). So far, this technology has been developed and tested exclusively in conditions where users interact with devices on a static table. Nonetheless, it is well-known that physical perturbations directly interfere with perceived tactile sensations due to undesirable masking effects, such as a reduced perceived intensity or not noticing the tactile stimulus (Jamalzadeh et al., 2021; Ryu et al., 2018; Vardar et al., 2018; Verrillo, 1985). Moreover, the rise in unintentional physical finger movements due to external perturbations causes fluctuations in finger contact force and scan speed, leading to variations in the finger contact area (Adams et al., 2013; Ayyildiz et al., 2018; Delhaye et al., 2014; Sahli et al., 2018), stick-slip behavior (Ozdamar et al., 2020), and the air gap between the touchscreen and the skin (AliAbbasi et al., 2023; Shultz et al., 2018; Vardar & Kuchenbecker, 2021). Such changes to the finger-touchscreen interface significantly affect generated tactile stimuli (e.g., forces) and what users feel (Vardar & Kuchenbecker, 2021). Understanding how these factors affect electrovibration forces and perception could be leveraged to counteract them, for example, by carefully adapting tactile stimuli based on the (measured) signal characteristics of the perturbations.

Here, we investigate the effect of whole-body vibrations in the vertical direction due to external vehicle perturbations (e.g., turbulence) on the perception of electrovibration displayed on touchscreens. For this goal, we conducted psychophysical experiments where we tested the detection thresholds of human participants for short- and long-duration electrovibration stimuli in the absence and presence of whole-body vibrations. Concurrently, we recorded the finger contact force, scan speed, and position. Finally, we analyzed how the external perturbations influenced the recorded finger contact forces, scan speeds, and positions and explained their possible effect on the measured thresholds.

### Hypotheses

We expect a shorter-duration electrovibration stimulus to be more challenging to feel, resulting in higher perceptual thresholds. Next, we anticipate that the whole-body vibrations (e.g., external vehicle perturbations) will negatively affect the perception of electrovibration, resulting in higher thresholds. Moreover, we expect that the vertical finger scan speed and displacement error, the applied normal force, and the rate of change in this force will increase due to unintentional limb movement resulting from vertical whole-body vibrations.

## Methods

To test our hypothesis, we measured the absolute detection thresholds of human participants for electrovibration stimuli, both in the presence and absence of whole-body vibrations (e.g., turbulence).

### Participants

The psychophysical experiments were conducted with seventeen male and seven female participants with an average age of 25.6 years and a standard deviation of 3.8 years. All participants were right-handed except one. The total number of participants was determined via power analysis to determine the number of participants, as detailed in (Cohen, 1992). The effect size was set to 0.25 based on the results of the first 18 participants. The significance level (*α*) was chosen as 0.05, and the statistical power was set to 1 − 4*α* = 0.8. Using these parameters, along with the number of groups and measurements, we used GPower 3.1 software to calculate the required sample size. The software suggested a minimum of 19 participants. To account for the use of a nonparametric test and to enhance the reliability of our experiment, we increased the sample size by 15%, resulting in 22 participants. Finally, to ensure homogeneous randomization across the six conditions (6 conditions × 4 = 24), we conducted the experiments with 24 participants.

### Experimental Setup

**Figure 1.**
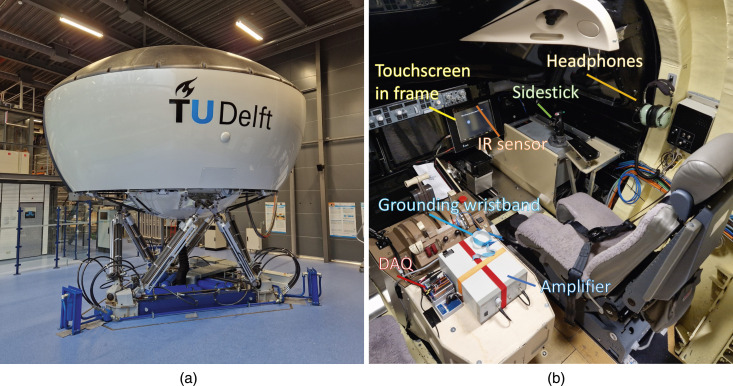
The experimental setup. (a) The outside view of SIMONA research simulator, (b) the simulator’s cabin with experimental equipment.

The experiments were conducted in the SIMONA Research Simulator at the Faculty of Aerospace Engineering of TU Delft; see Figure 1(a). The simulator’s 6 degrees-of-freedom hexapod motion system (Berkouwer et al., 2005) was used to generate vertical whole-body vibrations simulating aircraft turbulence.

During the experiments, the participant sat in the right seat of the simulator and was strapped in by the five-point harness; see Figure 1(b). In front of the participant, a touchscreen (SCT3250, 3M Inc) was mounted in a custom-made frame that covered the regular primary flight display (ProLite TF1534MC-B1X, Iiyama Corp). The touchscreen had an 18-degree angle to the vertical plane in this configuration with a touch area of 255 mm × 177 mm. The original flight display below the frame showed a graphical user interface to guide the participants during the experiments. Within the frame, the touchscreen was attached to four force sensors (FSG020WNPB, Honeywell Inc) placed at the corners via double-sided tape. These sensors measured the normal force exerted on the touchscreen by the user’s finger with a range and resolution up to 20 N and 0.0098 N, respectively. A data acquisition board (NI-9205, NI Inc) collected force sensor data at a sampling rate of 2 kHz. An infrared position sensor (NNAMC2300PCEV, Neonode Inc) mounted on the top edge of the touchscreen measured the participant’s finger position and velocity with a touch resolution of 0.1 mm, 10 ms response time, and 100 Hz scanning frequency. The frame encapsulated the edges of the touchscreen, the sensors, and the wiring to keep the setup in place while the simulator was moving and as a safety measure for the participants. The participants could adjust the seat position to be comfortable while interacting with the touchscreen.

The electrovibration stimuli were generated by applying voltage signals to the touchscreen; more details regarding stimuli are provided in the Stimuli subsection. These signals were generated through another data acquisition card (NI-9264, NI Inc) and then augmented by a high-voltage amplifier (HVA200, Thorlabs Inc). The infrared position sensor and the data acquisition card were connected via USB cable extenders to a computer outside the simulator, in the simulator’s control room. The participants wore an anti-static wristband on their nondominant wrist for grounding and a noise-canceling aviation headset (H10-66XL, David Clark Inc) with which they could communicate and hear instructions from the experimenter in the control room. During the experiment, the participants listened to aircraft engine noise to mask any auditory cues from the simulator’s motion system. The participants entered their responses through the side stick, see Figure 1(b); they used the red and green side buttons to select a stimulus interval and the trigger button in the front to initiate the start of a trial. This sidestick was on the right side of the seat, which meant all participants had to use their right hand to press the buttons and touch the screen. A delay of 1 s was implemented between pressing the button and starting the trial to avoid losing touch data.

### Stimuli

#### Target Stimulus (Electrovibration)

**Figure 2.**
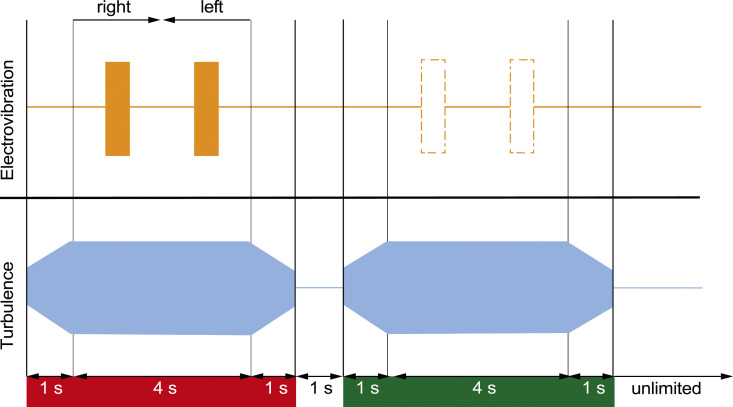
Example stimulus timing diagram for the absolute threshold experiments. The target (electrovibration) stimulus was generated by bursts of 100 Hz input voltage signals applied to the touchscreen. In threshold experiments with masked stimuli, the masking (turbulence) stimulus was whole-body vibrations with 1-s fade-in and fade-out. Both target and masking stimuli were displayed in two temporal intervals, which were signaled to the participants as red and green. The electrovibration stimulus was displayed randomly at either the red or green intervals. In the interval which did not have the electrovibration stimulus, the participants explored the smooth glass surface. In each interval, the participants explored the touchscreen in two strokes with a scan speed of 50 mm/s; each lasted 2 s. The participants gave their responses after the green interval ended.

The target stimulus was an oscillating electrostatic force (i.e., electrovibration) generated by applying a sinusoidal voltage signal at 100 Hz without a DC offset to the conductive layer of the touchscreen. As demonstrated in previous studies on electrovibration perception (Vardar et al., 2017, 2018; Vardar & Kuchenbecker, 2021), this input voltage generates an electrovibration stimulus at twice the input signal’s frequency, that is, 200 Hz, as the generated force is proportional to the square of the applied voltage; see Supplemental Materials. We selected this frequency as it is in the range of frequencies at which electrovibration is best perceived (Vardar et al., 2016). The stimulus amplitude varied throughout the experiment; see Procedure. The duration of the electrovibration stimulus was either 0.2 or 0.5 s, which was perceived twice in a trial that lasted 4 s by stroking the fingertip on the touchscreen back and forth in a left-right motion. The 0.2-s duration represented a rough duration of vibratory stimulation received when a finger passes over a 10 mm ridge (e.g., an edge or a small button) on the touchscreen. The 0.5-s stimulus represented interaction with a 25 mm feature, such as a small slider. Both stimuli were displayed such that their spatial center was aligned to the center of the touchscreen; see Figure 2 for a schematic view of a trial with a 0.5-s stimulus.

#### Masking Stimulus (Whole-Body Motion)

The masking stimulus consisted of mechanical vibrations generated by the SIMONA Research Simulator’s motion system, affecting the whole body of the participant while interacting with the touchscreen inside the cabin. We selected vibrations simulating vertical turbulence accelerations on a flying vehicle as the masking stimulus. We tested two different types of turbulence signals, here referred to as Multisine and Gaussian, which were previously designed and tested by Khoshnewiszadeh and Pool (2021) and Leto and Pool (2025) and had frequency components up to 10 Hz; see Supplemental Materials for details regarding their design. 

The original Gaussian and Multisine turbulence signals as used in (Leto & Pool, 2025) had a duration of 90 s. For the current experiment, 10 different 6-s segments of both signals were extracted for our stimulus interval (1-s fade-in, 4-s exposure, 1-s fade-out; see Figure 2. The 10 different masking stimuli were used to ensure participants could not learn and anticipate the whole-body motion stimuli after repeated exposure, thereby ensuring that a generalizable result was obtained for the electrovibration perception threshold. To ensure small differences between the 10 different motion stimuli did not affect the measured thresholds, the same motion stimulus was always used for both compared intervals at each staircase iteration (see Procedure).

### Procedure

Before the experiments, each participant washed their hands with water and soap and dried them at the natural room temperature. Then, they read and signed the informed consent form. Afterward, each participant watched a safety video for the SIMONA Research Simulator, and they were briefed about experimental procedures and warned about motion sickness. Then, each participant sat in the simulator and arranged the seat to interact with the touchscreen comfortably. While their final selected seat position ensured that each participant could comfortably interact (see and touch) with the touchscreen in the seated posture where their back was entirely in contact with the seat, they were free to change their posture during the experiments. The touchscreen was also cleaned with alcohol before each session. Each experimental session was started when the participant pressed the trigger button on the side stick.

The experiments aimed to determine the absolute detection thresholds of participants for the target (electrovibration) stimuli in the presence and absence of a masking stimulus (whole-body vibrations). The experiments followed the two-alternative-forced-choice (2AFC) method (Fencher, 1860). The stimuli were displayed in two temporal intervals; see Figure 2. These were signaled to the participant as red and green via a color-matched cursor on the graphical user interface (GUI); see Figure S1 for an example visual displayed in one interval. Each interval lasted for 4 s. Only one of those intervals contained the target stimulus, while an identical masking stimulus was present in both intervals. The location of the target stimulus was randomized for each trial and participant.

Each participant was instructed to hold the index finger of their dominant hand on the screen indicated with the virtual cursor and move in the horizontal direction while synchronizing their finger movements with the cursor’s motion, which moved with a speed of 50 mm/s (see Figure S1). This finger speed resulted in an exploration area of 100 mm wide. In each interval, participants performed a 2-s stroke to the right, immediately followed by a continuous 2-s stroke to the left, all while keeping continuous contact with the screen. After the red interval ended, they kept their finger at the starting point for 2 s and then repeated the same procedure in the green interval.

After experiencing the two intervals, the participant’s task was to indicate whether the target stimulus was in the red or green interval. While they could not repeat the intervals, they had unlimited time to respond to the stimuli they experienced in that trial. They registered their choice by pressing the red or green buttons on the sidestick. They pushed the sidestick’s trigger button when ready for the subsequent trial.

The amplitude of the target stimulus was modified via the three-up/one-down adaptive staircase method (Levitt, 1971). Each session started with a stimulus generated by a (peak) input voltage of 50 V. This amplitude was chosen based on preliminary experiments and participant training sessions to ensure that all participants clearly felt the initial stimulus. If the participant gave three correct answers (not necessarily consecutive), the voltage amplitude was decreased by 5 dB. If the participant gave one incorrect answer, the amplitude was increased by 5 dB. The change from increasing intensity to decreasing and vice versa is called reversal. After one reversal, the step size was changed to 1 dB. The session ended after five reversals in a ± 1 dB level, and the mean value of these last five reversal voltage levels was taken as the absolute threshold. The dB unit used in research on electrovibration is described as 20 log_10_ (*V*_*p*_) with *V*_*p*_ being the peak voltage of the touchscreen. The maximum peak amplitude of the applied voltage was set at 100 V. For all participants, the whole staircase was completed in approximately 30–50 trials.

Each participant completed the experiments in six sessions: 2 test signal durations (0.2 and 0.5 s) × 3 whole-body vibration cases (no motion, Multisine, and Gaussian motion). The sessions were conducted in six different orders between participants to balance out order effects. Within all whole-body vibration sessions, the turbulence signal for each staircase trial was a randomized choice from the ten 6-s stimuli that were available, see Stimuli. To ensure an unbiased comparison between the two intervals of each staircase trial, the exact same turbulence stimulus was always used for both intervals (see Figure 2).

From the collected data, the measured absolute threshold voltages, average finger speeds, average applied forces, and average rate of change in applied forces were extracted; see Supplemental Materials for details on signal processing. For the statistical analysis, we first checked the normality of the extracted data using Shapiro–Wilk tests. As one or more samples were not normally distributed for all data types, we used nonparametric Friedman ANOVAs to test the main effects. Then, we applied Bonferroni-corrected Wilcoxon signed rank tests for posthoc pairwise comparisons. All data are depicted in box plots in this paper, with the results for the 0.2-s and 0.5-s electrovibration stimuli shown in green and yellow, respectively. The center red lines signify medians, while the box limits delineate the 25th and 75th percentiles. Whiskers extend to 1.5 times the interquartile range. Data outside of 1.5 times the interquartile range are denoted as outliers and indicated by red plus signs (+), while yellow diamonds (⋄) signify sample means. All data, including the outliers, were considered in the statistical analyses. Circles represent (◦) individual measurements from each participant. Overbraces with indicated asterisks indicate statistically significant differences (* for *p <* 0.05 and ** for *p <* 0.001).

## Results

**Figure 3.**
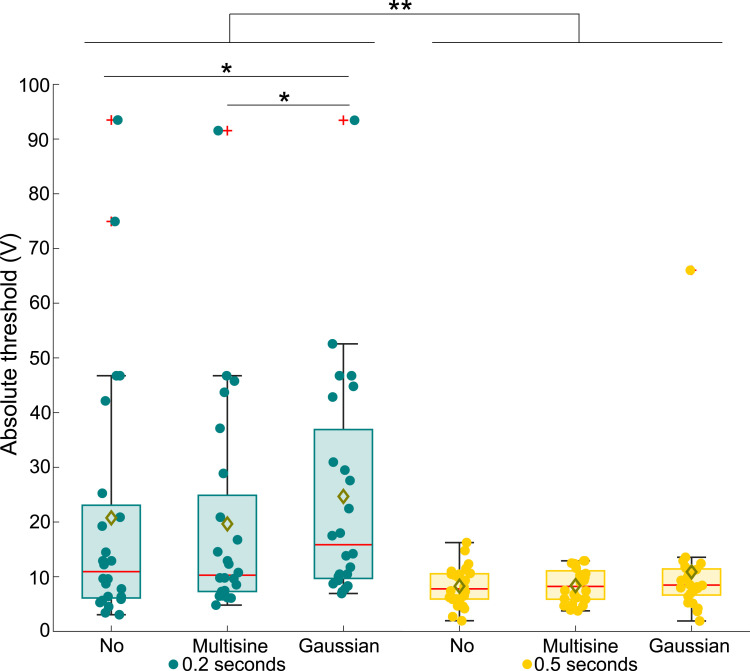
Absolute threshold voltages for all tested experiment conditions.

**Figure 4.**
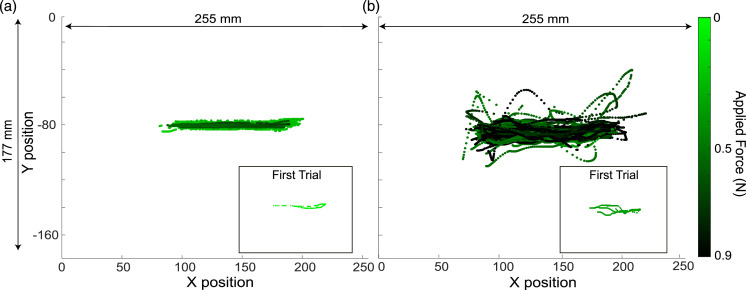
Measured finger positions of a participant during two complete experiments with 0.5-s electrovibration stimuli during (a) no-turbulence (47 trials) and (b) Multisine turbulence (37 trials). The corresponding applied normal force is shown as a heat map. The first trial of each condition is highlighted within a scaled rectangle.

**Figure 5.**
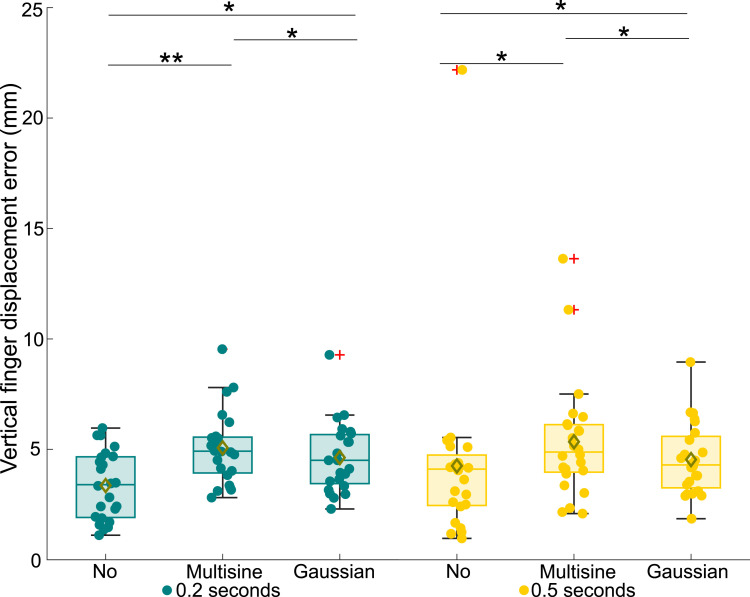
Average finger displacement error in the vertical direction of participants for all tested experiment conditions.

**Figure 6.**
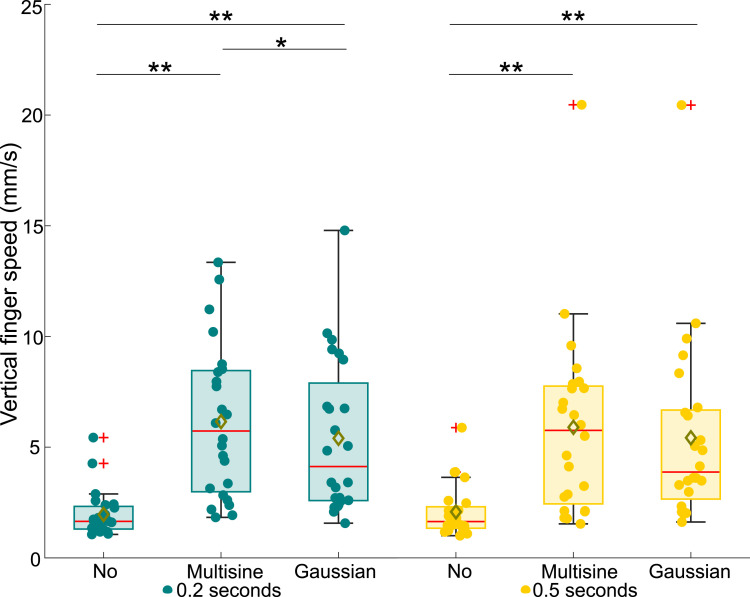
Average finger speeds of participants in the vertical direction for all tested experiment conditions.

**Figure 7.**
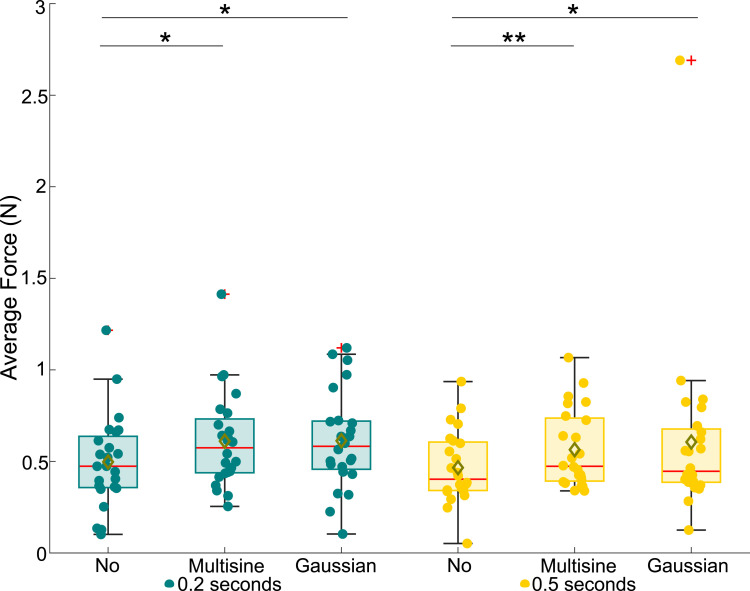
Average applied forces of participants for the tested experiment conditions.

**Figure 8.**
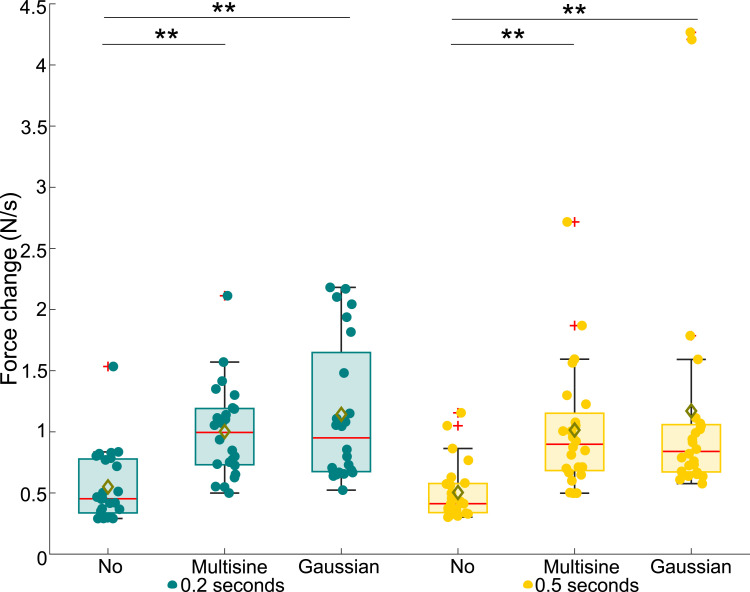
Average rate of change in the average applied forces of participants across different experimental conditions.

The absolute threshold voltages measured for the participants are presented in Figure 3. As shown, the distributions for the 0.2-s electrovibration stimulus, both without turbulence and with the turbulence conditions (labeled as “Multisine” and “Gaussian” in Figures 3–8), exhibit very high outliers (>90 V). These outliers suggest that the corresponding participants could not reliably perceive the stimulus. Overall, a statistically significant difference in the absolute threshold across all test cases was found *χ*^2^ (5) = 45.53, *p <* 0.001, as well as a highly significant effect of electrovibration duration (*χ*^2^ (1) = 36.63, *p <* 0.001). Across all turbulence conditions, the mean thresholds and variances were greater for the 0.2-s stimulus; see Figure 3. While no significant effect of turbulence condition was observed for the 0.5-s stimulus (*χ*^2^ (2) = 2.8, *p >* 0.05), a significant effect of turbulence was found for the 0.2-s stimulus (*χ*^2^ (2) = 8.49, *p <* 0.05). Pairwise comparisons also showed a significant difference between the no-vibration and Gaussian vibration conditions (*p <* 0.05), as well as between the Multisine and Gaussian vibration conditions (*p <* 0.05).

Figure 4 shows an example finger position measurement (34 aggregated trials) from one participant in the absence and presence of whole-body vibrations (compare Figures 4(a) and (b)). The figure axes match the geometry and aspect ratio of the touchscreen, showing the participant trying to follow a line in the center of the screen where the cursor was moving. The effect of unintended finger motions due to the whole-body vibrations is visible, with an almost straight horizontal finger trajectory during no-turbulence and large undesired vertical movements during the Multisine vibration. Figure 4 also shows that the applied force is higher in the presence of whole-body vibrations compared to when they are absent. An example of recorded raw force data can be found in Figure S2.

We analyzed the average displacement error in the vertical direction measured for the different experiment conditions; see Figure 5. The displacement error in the vertical direction varied significantly across conditions (*χ*^2^ (5) = 28.81, *p <* 0.001). Specifically, turbulence significantly increased the displacement error for both stimulus durations: 0.2 s (*χ*^2^ (2) = 18.08, *p <* 0.001) and 0.5 s (*χ*^2^ (2) = 9.75, *p <* 0.05). Pairwise comparisons revealed significant differences between the no-turbulence and turbulence conditions for each stimulus duration (*p <* 0.05).

The average finger speeds in the vertical direction measured for the different experiment conditions are shown in Figure 6. Overall, a highly significant variation in vertical finger speed was found across all tested conditions (*χ*^2^ (5) = 69.76, *p <* 0.001). We found that turbulence significantly increased the vertical finger speed for both stimulus durations (0.2 s: *χ*^2^ (2) = 36.58, *p <* 0.001, 0.5 s: *χ*^2^ (2) = 25.75, *p <* 0.001), whereas the target stimulus duration did not have an effect (*χ*^2^ (1) = 1.39, *p >* 0.05). Pairwise comparisons showed that there was a significant difference between the no-turbulence and the turbulence conditions for both stimulus durations (*p <* 0.001), but also between the two different turbulence conditions for the 0.2-s stimulus (*p <* 0.05). We also analyzed the average horizontal finger speeds but found no significant effect of either vibration type or electrovibration stimulus duration.

Figure 7 shows the average normal force applied on the touchscreen during the experiments. The overall Friedman ANOVA showed a significant effect across all tested conditions (*χ*^2^ (5) = 15.64, *p <* 0.05). Furthermore, we found a significant effect of vibration on the average force for both the 0.2-s (*χ*^2^ (2) = 9.08, *p <* 0.05) and 0.5-s (*χ*^2^ (2) = 18.58, *p <* 0.001) target stimuli, but no main effect of stimulus duration (*χ*^2^ (1) = 0.89, *p >* 0.05). In pairwise comparisons, only the no-turbulence condition was significantly different from both turbulence conditions across both stimulus durations (*p <* 0.05).

The calculated rate of change in applied force for different experimental conditions is shown in Figure 8. The average force change varied significantly across all experiment conditions (*χ*^2^ (5) = 77.95, *p <* 0.001). Furthermore, we found a significant effect of turbulence on the rate of change in the measured average force for both the 0.2-s stimulus (*χ*^2^ (2) = 28.08, *p <* 0.001) and the 0.5-s stimulus (*χ*^2^ (2) = 36.00, *p <* 0.001). Pairwise comparisons showed that this increase in average force change was not significantly different between the Multisine and Gaussian turbulence cases for either of the stimulus durations (0.2 or 0.5 s). However, there was no significant effect of stimulus duration (*χ*^2^ (1) = 3.56, *p >* 0.05). Moreover, pairwise comparisons showed no statistically significant differences for the individual turbulence conditions.

Notably, all results remained consistent between the initial 18 participants and the complete set of 24 after the power analysis, except for the effect of stimulus duration on the force change. Specifically, this effect was statistically significant (*p <* 0.05) with 18 participants, but lost its significance (*p >* 0.05) when the extended sample was analyzed.

## Discussion and Conclusions

This study investigates the influence of whole-body vibrations, such as aircraft turbulence, on the perception of electrovibration displayed on touchscreens. For this objective, we measured the absolute detection thresholds of 24 human participants for electrovibration stimuli with varying durations, both in the absence and presence of two types of whole-body vibrations. Concurrently, we measured participants’ applied normal force and finger speed during the experiments. We hypothesized that the detection thresholds for shorter-duration electrovibration stimuli would be higher than those for longer durations. Furthermore, we anticipated an increase in detection thresholds, finger speed, and displacement error in the vertical direction, as well as the applied normal force and the rate of change of this force when whole-body vibrations were introduced.

The first hypothesis, predicting an increase in absolute thresholds for shorter electrovibration stimulus duration, is supported by the significantly higher thresholds observed with shorter electrovibration durations compared to longer ones (see Figure 3, green and yellow box plots). Additionally, the higher variance in measured thresholds for the 0.2-s stimuli suggests that 0.5-s stimuli were perceived more consistently by participants. This outcome can be attributed to a temporal summation, that is, the integration of energy over time by a sensory system. Previous studies have reported similar results using 200 Hz vibrotactile stimuli (Checkosky & Bolanowski, 1994; Geschieder & Joelson, 1982).

Contrary to the second hypothesis suggesting an increase in absolute thresholds due to turbulence, we only found significantly elevated measured thresholds with turbulence for the 0.2-s electrovibration stimulus (see Figure 3). This outcome suggests that this threshold increase is not attributable to perceptual masking, as such an effect would impact both stimulus durations. The considerable frequency difference between the turbulence and electrovibration stimuli may explain the lack of perceptual masking due to turbulence vibrations. Previous research also supports this claim by demonstrating that mechanical vibration influences electrovibration perception predominantly when both stimuli share similar frequencies (Jamalzadeh et al., 2019; Ryu et al., 2018).

The significantly elevated thresholds with turbulence for shorter electrovibration stimuli can be attributed to significant force changes during turbulence (Figure 8) combined with more unintentional finger movements (see Figures 4–6). Participants likely often missed the short 0.2-s stimulus during turbulence, for example, as in the case of simultaneous normal force, finger speed, and displacement variations. In contrast, a more extended stimulus duration gave participants greater chances to perceive the electrovibration despite sudden touch interaction changes. Furthermore, several participants commented about their fingers “jumping” over the screen during turbulence, supporting this notion. Unfortunately, these ‘jumps’ could not be reliably extracted from the IR or force sensor signals due to high noise levels. Future research could employ electrical impedance measurements to detect such instances of loss-of-contact (Forte et al., 2024; Vardar & Kuchenbecker, 2021).

Our hypothesis asserting that the vertical finger scan speed and displacement error (in the direction of turbulence motion) would significantly increase during turbulence is confirmed (see Figures 5 and 6). The increase from average finger speeds around 2 mm/s to around 6 mm/s, caused by additional unintentional finger movements as a result of the applied vertical whole-body vibration, is consistent with earlier studies (Khoshnewiszadeh & Pool, 2021; Mobertz et al., 2018).

Lastly, the hypothesis predicting that turbulence would significantly increase the applied normal force and its rate of change is also supported by our data; see Figures 7 and 8. The elevated normal force during turbulence likely results from participants deliberately compensating to prevent unintentional sliding motions caused by the turbulence. This adjustment led participants to exert greater steady-state and dynamically changing force on the touchscreen (Khoshnewiszadeh & Pool, 2021; Venrooij et al., 2013).

Interestingly, despite the significant changes in the finger contact dynamics (e.g., changes in contact force and finger speed) during turbulence, there were no substantial alterations in the perceptual thresholds for the long-duration electrovibration stimulus. Nonetheless, past research has shown that changes in finger speed and applied normal force affect finger contact area, the air gap between the touchscreen, and the stick-slip behavior of the fingertip, directly influence the generated electrostatic force and its perception (Vardar et al., 2017; Vardar & Kuchenbecker, 2021). Some of these conditions are known to adversely affect each other, such as increased applied force leading to increased contact area (Ayyildiz et al., 2018), but increased stick-slip behavior and a decreased air gap; consequently, the overall effect could hide such underlying changes. A more detailed investigation of how these factors dynamically change during turbulence is needed (Pool & Vardar, 2024), also taking into account that these physical factors regarding finger contact dynamics are known to vary greatly between people (Nam et al., 2020; Serhat et al., 2022). Finally, in our experiment, the electrovibration stimulus was presented in a time-based manner; hence, it could be perceived anywhere on the screen, regardless of the actual finger movement. For the more realistic presentation of the electrovibration only at a fixed location on the graphical interface, the effects of turbulence motion are likely more severe. We will test this hypothesis in our future work.

Despite our careful investigation, this study had some limitations. For instance, the seating posture of the participants was not restricted. Participants whose backs were not entirely in contact with the seat may have experienced higher propagated vibrations. Moreover, this situation might have caused variations in the user’s touchscreen viewing angle. Additionally, the left-handed participant entered responses using the hand, not engaged in exploring the screen, which differed from the approach of right-handed participants.

Our results offer valuable insights for the future design of touchscreen interactions in vehicle cockpits. Electrovibration-rendered virtual buttons or sliders should be substantial enough to generate stimuli representing graphical features larger than 1 cm (approximately 0.2-s stimulation duration) to reduce susceptibility to turbulence. Additionally, fluctuations in finger speed and normal force caused by turbulence likely need to be actively compensated for in tactile rendering to maintain consistent electrovibration intensity and ensure reliable user interaction.

## Key Points

• Electrovibration stimuli displayed on vehicle cockpit touchscreens are more reliably perceived with a 0.5-s duration than a 0.2-s duration, both with and without turbulence.
• Low-frequency whole-body vibrations, such as turbulence impair the perception of short (0.2-s) electrovibration stimuli, but not longer ones (0.5-s).
• Perceptual masking does not occur for electrovibration stimuli in the presence of low-frequency whole-body vibrations, such as aircraft turbulence.
• Unintentional arm movements due to turbulence vibrations increase the finger scan speed and displacement error, the average applied force, and the rate of force change.

## Supplemental Material

Supplemental Material - Impact of Whole-Body Vibrations on Electrovibration Perception Varies with Target Stimulus Duration Supplemental Material for Impact of Whole-Body Vibrations on Electrovibration Perception Varies with Target Stimulus Duration by Jan D. A. Vuik, Daan M. Pool, Celal Umut Kenanoglu, and Yasemin Vardar in Human Factors.
