# Supplementary material for: Impact of Whole-Body Vibrations on Electrovibration Perception Varies with Target Stimulus Duration
Source: Hum Factors. 2025 Apr 17;67(10):1046–61. doi: 10.1177/00187208251326662 (PMC12420938; doi:10.1177/00187208251326662)
Supplement: Supplemental Material - Impact of Whole-Body Vibrations on Electrovibration Perception Varies with Target Stimulus Duration [file sj-pdf-1-hfs-10.1177_00187208251326662.pdf]

# Supplementary materials for “Impact of whole-body vibrations on electrovibration perception varies with target stimulus duration”

Jan D. A. Vuik[1], Daan M. Pool[2], Celal Umut Kenanoglu[1], and Yasemin Vardar[1]

[1]Department of Cognitive Robotics, Faculty of Mechanical Engineering, Delft University of Technology, Delft, The Netherlands

[2]Department of Control and Operations, Faculty of Aerospace Engineering, Delft University of Technology, Delft, The Netherlands

# Supplementary materials for “Impact of whole-body vibrations on electrovibration perception varies with target stimulus duration”

## Electrovibration Technology

Electrovibration is a technology that generates tactile feedback on touchscreens by modulating interaction forces between the screen surface and the user’s finger through electrostatic actuation (Basdogan et al., 2020). This approach provides a rapid, dynamic, and high-bandwidth modulation that is scalable, noise-free, and easy to implement technique for enhancing future touchscreen interfaces.

When an alternating voltage is applied to the touchscreen’s conductive layer, a periodic electrostatic attractive force is generated between the user’s finger and the screen (Bau et al., 2010). This force arises from opposite charges induced on the screen’s insulating layer and on the user’s finger. The outer tissue layer of the finger (stratum corneum) and the air gap between the finger and screen also act as insulators, while the fingertip’s inner tissue serves as a conductor. As the user moves their finger across the screen, the periodic attractive force creates a varying frictional force, producing tactile sensation. The magnitude of the electrostatic attraction force can be calculated as (Basdogan et al., 2020; Vodlak et al., 2016)

$$F_e = \varepsilon_0 \frac{AV^2}{2\left(\frac{d_{ins}}{\varepsilon_{ins}} + \frac{d_{air}}{\varepsilon_{air}} + \frac{d_{SC}}{\varepsilon_{SC}}\right)^2 \varepsilon_{air}} \tag{1}$$

where $A$ denotes the contact area of the fingerpad; $d_{ins}$, $d_{SC}$, and $d_{air}$ represent the thicknesses of the touchscreen’s insulating layer, the stratum corneum, and the air gap between the touchscreen and finger, respectively. Correspondingly, $\varepsilon_{ins}$, $\varepsilon_{SC}$, and $\varepsilon_{air}$ are their relative permittivities, and $\varepsilon_0$ is the permittivity of free space. Hence, the generated electrostatic actuation force depends on the contact area during interaction and the air gap between the finger and touchscreen, while other parameters remain constant for a given touchscreen.

The electrostatic force depends on the square of the applied voltage, as shown in Eq. 1. For a sinusoidal voltage $V = V_0 sin(\omega t)$ with no DC offset, squaring the sine wave produces

$V_0^2 \frac{1-cos(2\omega t)}{2}$ as derived from the trigonometric identity. This illustrates the frequency-doubling effect, a direct result of the nonlinearity in the force-voltage relationship.

## Design of masking stimuli

Both turbulence signals were designed for an RMS vertical acceleration of 0.75 m/s$^2$, representative of heavy turbulence (Coutts et al., 2019; Leto & Pool, 2025).

The Multisine turbulence signal was identical to those used in previous experiments (Khoshnewiszadeh & Pool, 2021; Mobertz et al., 2018) and defined as a sum of ten sinusoids:

$$\sum_{k=1}^{10} A_k \sin(\omega_k t + \phi_k), \tag{2}$$

where $A_k$, $\omega_k$, and $\phi_k$ represent the amplitude, frequency, and phase offset of each sinusoid. The frequencies $\omega_k$ ranged from 0.06 Hz (0.383 rad/s) to 2.76 Hz (17.33 rad/s). For the Multisine vibrations, 50% and 100% of the vertical acceleration RMS is due to components below 1.67 Hz and 2.76 Hz, respectively, i.e., much lower frequencies than the 100 Hz of our target stimulus.

The Gaussian turbulence represented the vertical accelerations of a simulated Cessna Citation 500 business jet (Van Der Linden, 1996) flying through stationary turbulence, modeled using Dryden spectra (Leto & Pool, 2025; Van de Moesdijk, 1978). For the Gaussian turbulence, 50% of the vertical acceleration RMS is due to components below 0.52 Hz, i.e., more low-frequency power compared to the Multisine turbulence. Furthermore, 96.4% of the signal's power was at frequencies below 2.76 Hz (the maximum sinusoid frequency for the Multisine turbulence). To protect the motion platform's hardware from undesired low-amplitude high-frequency vibrations, the Gaussian turbulence signal was filtered with a second-order low-pass filter with a cut-off frequency of 10 Hz.

## Data analysis

We stored the data acquired from the experiment in one file per staircase and analyzed it with a Matlab program. The force and finger position data consisted of a time series per staircase, with parts of the breaks present. We extracted the trial data from these time series by taking only the data from the start and finish of every interval. Any data from either the breaks between trials

or the breaks between intervals was omitted. The force data was recorded at 2000 Hz, the maximum achievable data rate of the sensors. The finger position data was recorded at 100 Hz. After calibrating the force sensors, we calculated the average applied normal force using the force from every sensor and the finger location to make a weighted average. A zero-phase digital second-order low-pass Butterworth filter filtered the force data to reduce the effect of high-frequency measurement noise, especially for the force change signal (time derivative of the force measurement). The finger location data was converted from pixels to millimeters, after which the data points outside the screen were filtered out. Then, the x- and y-location time series were differentiated to obtain the finger speed in both directions. In this data, values higher than 1000 mm/s, which corresponded to instances of loss of contact with the screen, were filtered out.

The vertical displacement of the finger is calculated by subtracting the finger's initial position from its current position, as the cursor's vertical position on the screen remains constant throughout the entire stimulus duration.

## Supplementary Figures

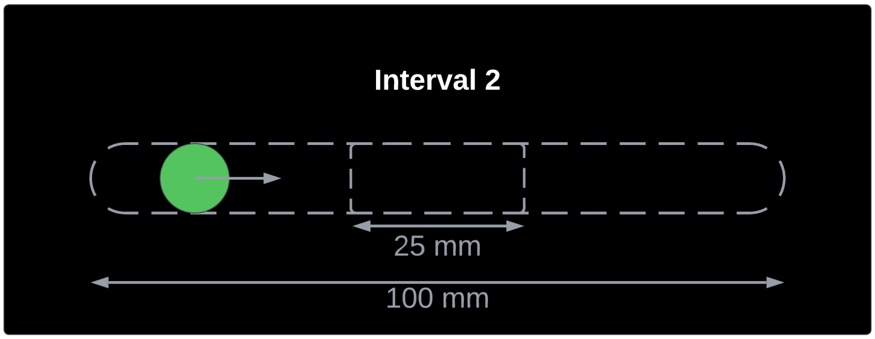


**Figure S 1**

*An example visual displayed on the screen during the second interval shows a green cursor and the text "Interval 2." Gray dashed lines, which indicate the 0.5-second interval duration, were present but not visible to the participant.*

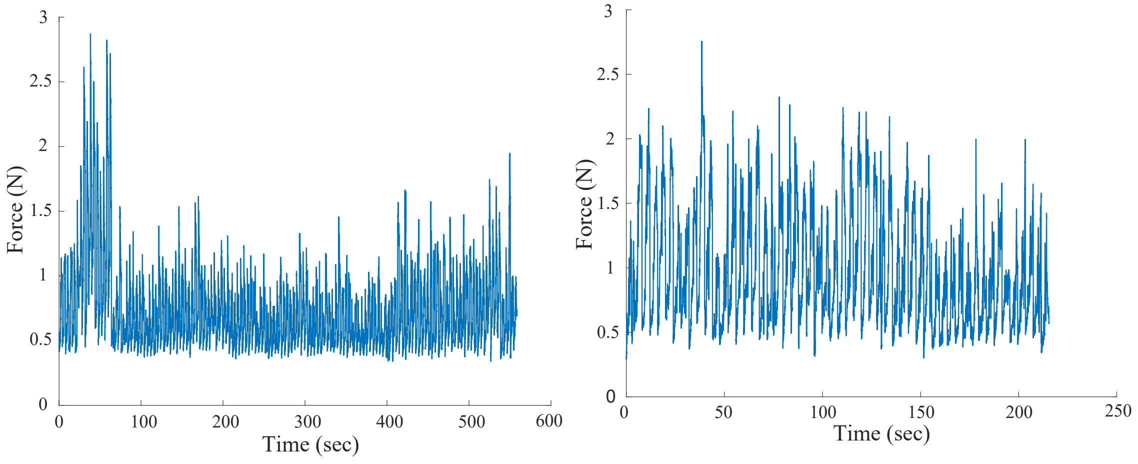


(a) (b)

**Figure S 2**

*Measured raw applied force data of a participant during two complete experiments with 0.5-second electrovibration stimuli during (a) no turbulence and (b) multisine turbulence.*
